# Effect of oliceridine pretreatment on etomidate-induced myoclonus: a prospective, randomized, double-blind, controlled study

**DOI:** 10.3389/fmed.2026.1764143

**Published:** 2026-03-06

**Authors:** Qingqing Sun, Xiaoqian Wang, Ziyuan Chen, Guimin Dong, Xinyuan Shi, Shiyu Yu, Hongyi Xiao, Fanceng Ji

**Affiliations:** 1 Department of Anesthesiology, Weifang People's Hospital, Weifang, China; 2 School of Anaesthesiology, Shandong Second Medical University, Weifang, China

**Keywords:** anesthesia induction, etomidate, myoclonus, oliceridine, pretreatment

## Abstract

**Purpose:**

Etomidate often induces adverse effects such as myoclonus during anesthesia induction, thereby increasing perioperative risks for patients to some extent. Oliceridine is a novel opioid with fewer opioid-related adverse reactions. This study aims to investigate the effect of oliceridine pretreatment on etomidate-induced myoclonus.

**Patients and methods:**

This study is a prospective, randomized, double-blind, controlled study. Patients scheduled for elective surgery under general anesthesia were selected and randomly divided into the oliceridine group (Group O) and the normal saline group (Group C), with 45 patients in each group. Two minutes before etomidate administration, Group O and Group C were given 0.02 mg/kg oliceridine and an equal volume of normal saline, respectively. Primary outcome measure: the incidence of etomidate-induced myoclonus. Secondary outcome measures: the severity of etomidate-induced myoclonus, as well as adverse reactions and hemodynamic changes occurring during the observation period.

**Results:**

The incidence of myoclonus in Group O was significantly lower than that in Group C (13.3% vs. 51.1%, RR = 0.45, 95% confidence interval [95%CI] = 0.310–0.667, *p* < 0.001). Compared with Group C, the incidence of myoclonus in Group O was reduced by 37.8%. Among the secondary outcomes, the severity of myoclonus in Group O was significantly lower than that in Group C (*p* < 0.001). The time to loss of consciousness in Group O was shorter than that in Group C (43.24 s ± 6.89 vs. 48.2 s ± 10.34, *p* = 0.008), and there was a statistically significant difference in the BIS values between the two groups at 2 min after etomidate induction (T2) (44.23 ± 9.38 vs. 53.75 ± 16.54, *p* = 0.001). In addition, there were no statistically significant differences in adverse reactions or hemodynamic changes between the two groups during the observation period.

**Conclusion:**

Pretreatment with oliceridine can significantly reduce the incidence of etomidate-induced myoclonus. Therefore, oliceridine can be used as a new pretreatment strategy when etomidate is employed for anesthetic induction.

**Clinical trial registration:**

https://www.chictr.org.cn/searchproj.html, identifier ChiCTR2500108944.

## Introduction

Etomidate is a non-barbiturate intravenous anesthetic. Due to its mild respiratory and circulatory depression, it has become one of the drugs of choice for anesthesia induction (1). However, etomidate often induces adverse reactions such as myoclonus during the induction process, with an incidence rate as high as 50–80% (2, 3). This myoclonus often manifests as involuntary muscle twitching, tremors, or tonic–clonic movements. Although self-limiting, it increases perioperative risks to some extent and, in severe cases, may lead to regurgitation and aspiration (4, 5).

Oliceridine is a novel opioid analgesic, classified as a G-protein-biased μ-opioid receptor agonist. It selectively activates the G-protein signaling pathway while significantly reducing activation of the β-arrestin pathway (6). While retaining analgesic efficacy, it can significantly reduce rather than eliminate opioid-related adverse reactions (7). Studies have shown that compared with sufentanil, a traditional opioid, oliceridine result in a lower incidence of respiratory depression and higher safety (8). Currently, there are no relevant studies on oliceridine pretreatment. Therefore, this study intends to explore the effect of oliceridine pretreatment on etomidate-induced myoclonus, so as to provide a reference for optimizing the etomidate medication regimen.

## Materials and methods

### Study design and patient enrollment

This study was approved by the Weifang People’s Hospital (Ethics Approval Number: KYLL20250829-1) and registered with the Chinese Clinical Trial Registry^1^ (Registration number: ChiCTR2500108944, 09/09/2025). This study adhered strictly to the Declaration of Helsinki and complied with the CONSORT guidelines (9). Prior to enrollment, all patients provided written informed consent.

This study is a prospective, randomized, double-blind, controlled trial. Patients scheduled for elective surgery under general anesthesia between September 2025 and October 2025 were selected. The inclusion criteria were as follows: aged 18–65 years, ASA physical status I–II, and body mass index (BMI) of 18–28 kg/m^2^. Exclusion criteria included: patients with adrenal cortex dysfunction; patients with hepatic or renal insufficiency; presence of difficult airway; potential allergy to the study-related medications; patients with psychiatric or cognitive impairments; and patients who had received sedatives/anesthetics within 24 h prior to anesthesia induction.

A researcher who only participated in randomization assigned patients to either the oliceridine group (Group O) or the normal saline group (Group C) at a 1:1 ratio using the random number table method, with 45 patients in each group. The randomization results were sealed in sequentially numbered envelopes, and personnel not involved in data collection prepared the study medications according to the grouping specified in the envelopes. The oliceridine to be pre-injected was calculated, diluted to 5 mL, and stored in 5-mL syringes; normal saline was also prepared as 5-mL aliquots and stored in 5-mL syringes. All syringes were labeled as “preinjection medication” to ensure that the anesthesiologists were unaware of the grouping. In addition, neither the patients nor the researchers responsible for postoperative follow-up were aware of the grouping.

### Anesthesia management

Patients were routinely fasted before surgery and without premedication. After the patient enters the operating room, monitor vital signs including non-invasive blood pressure, pulse Oxygen Saturation (SpO_2_), electrocardiogram, and bispectral Index (BIS). After 5 min of pre-oxygenation via the patient’s face mask, group O and group C were administered 0.02 mg/kg of oliceridine and an equal volume of normal saline, respectively. Patients were observed for 2 min to assess for any discomfort.

Anesthesia induction: Each patient was administered 0.3 mg/kg of etomidate via intravenous infusion over 30 s. After etomidate administration, patients were observed for 2 min to assess the occurrence and severity of myoclonus. Simultaneously, the time to loss of consciousness was recorded, with the disappearance of the eyelash reflex used as the indicator for loss of consciousness.

At the end of the observation period, 0.6 mg/kg of rocuronium and 0.3 μg/kg of sufentanil were administered. After waiting 2 min for the muscle relaxant to take effect, an appropriately sized endotracheal tube was inserted. Mechanical ventilation was initiated to maintain respiration, and anesthesia was maintained with sevoflurane and remifentanil until the conclusion of the surgery. During the research process, if a patient’s systolic blood pressure fell below 90 mmHg, 5 μg of norepinephrine was administered; if the heart rate dropped below 50 beats per minute, 0.5 mg of atropine was given as intervention.

### Assessment of primary and secondary outcomes

The primary outcome measure was the incidence of etomidate-induced myoclonus. The secondary outcome measures were as follows: 1. The severity of etomidate-induced myoclonus, which was classified into three grades: mild (movement limited only to the fingers or wrists); moderate (mild movement in a specific part of the body, such as the face or legs); severe (generalized body response or rapid abduction of limbs). 2. Adverse reactions occurring during the period from the administration of oliceridine or normal saline to the end of the etomidate observation period, including dizziness, nausea, respiratory depression (defined as SpO_2_ < 95%), bradycardia (defined as heart rate below 50 beats per minute), hypotension (defined as systolic blood pressure below 90 mmHg), increased secretions, and coughing. 3. Hemodynamic changes during the induction phase: the systolic blood pressure (SBP), diastolic blood pressure (DBP), mean arterial pressure (MAP), heart rate (HR), and BIS of patients were observed and recorded at three time points, namely when the patient entered the operating room (T0), 2 min after oliceridine administration (T1), and 2 min after etomidate induction (T2).

### Statistical analysis

The primary objective of this study was to observe the incidence of etomidate-induced myoclonus. Pilot studies demonstrated that the incidence of etomidate-induced myoclonus was 64%, and it was hypothesized that a reduction of more than 30% in the incidence of myoclonus was clinically significant (10). With settings of *α* = 0.05, 1 − *β* = 0.8, and a 1:1 ratio between the two groups, sample size calculation was performed using PASS software Version 2021, which determined that 40 patients were required per group. Considering an anticipated dropout rate of 10%, the planned enrollment was 45 patients per group.

All collected data were tested for normality using the Shapiro–Wilk test. Measurement data such as age, weight, and height, which conform to a normal distribution, are presented as mean ± standard deviation. Intergroup comparisons were analyzed using independent samples *t*-test. The incidence of myoclonus and other categorical data are presented as percentages, with intergroup comparisons analyzed using the chi-square test or Fisher’s exact test. *p* < 0.05 was considered statistically significant. All analyses and graphs were generated using R (Version 4.2.1, R Foundation for Statistical Computing, Vienna, Austria) and GraphPad Prism 7.0 (GraphPad Software Inc., San Diego, CA, USA).

## Results

**Figure 1:**
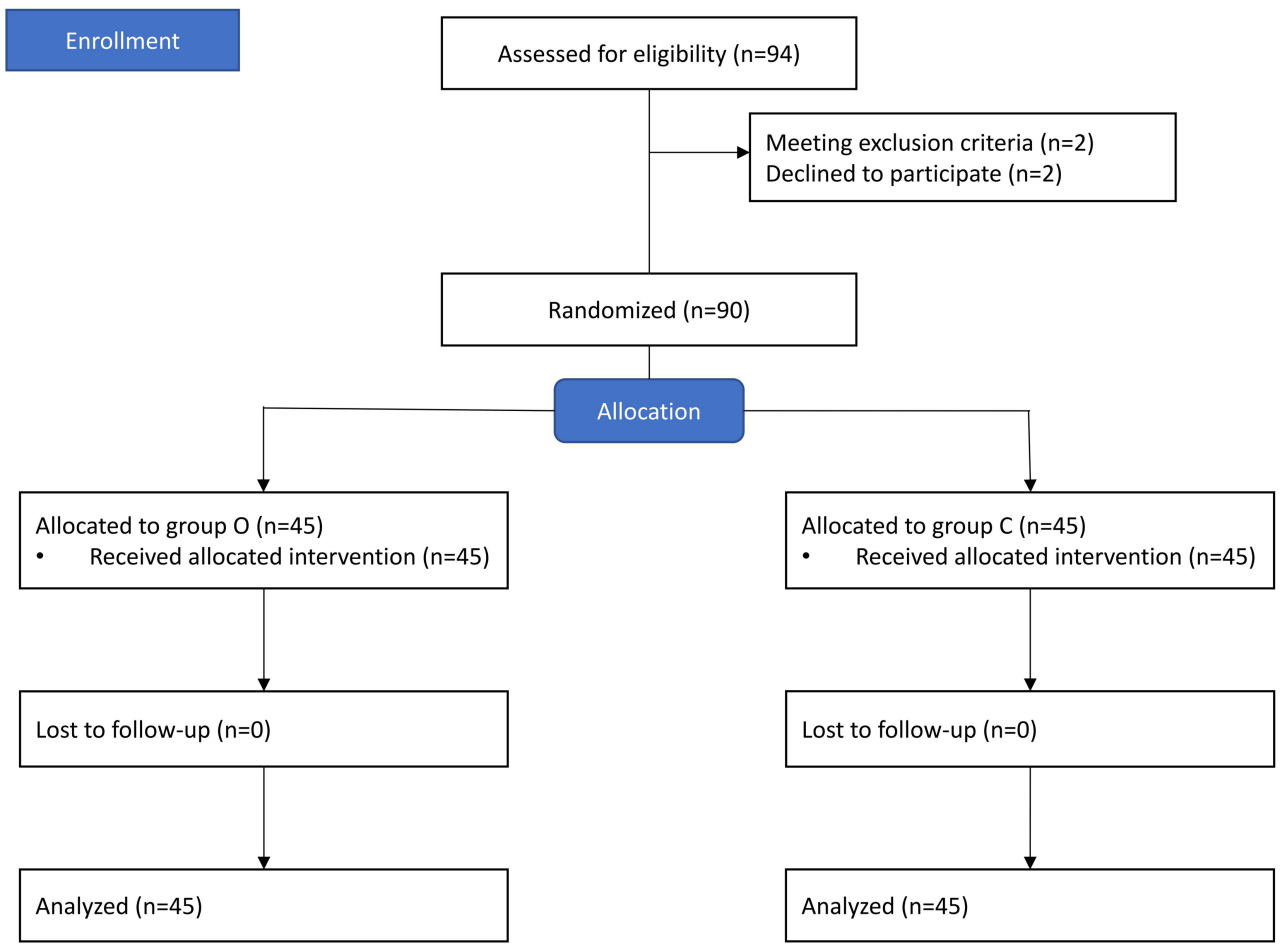
Study flowchart.

A total of 94 patients were screened for this study. Among them, two patients were excluded due to suspected difficult airway, and two patients refused to participate. Consequently, 90 patients were randomly assigned to either Group O or Group C, with 45 patients in each group (Figure 1).

Among the 90 patients included in the study, the mean age was 45.37 ± 13.14 years. No statistically significant differences were observed in the baseline demographic and intraoperative characteristics between the two groups (Table 1).

**Table 1:** Baseline demographic and intraoperative characteristics of the patients.

Variable	Group O (*n* = 45)	Group C (*n* = 45)	*p*-value
Age (years)	43.67 ± 12.52	47.07 ± 13.67	0.222
Weight (kg)	67.47 ± 10.20	64.66 ± 9.72	0.186
Height (cm)	165.31 ± 7.79	165.53 ± 8.04	0.894
BMI (kg/m^2^)	24.60 ± 3.07	23.58 ± 2.92	0.110
Sex (Male)	19 (42.2)	20 (44.4)	0.832
ASA [*n*, (%)]			1.000
I	4 (8.9)	3 (6.7)	
II	41 (91.1)	42 (93.3)	1.000
Etomidate (mg)	20.00 ± 3.05	19.28 ± 2.91	0.250
Oliceridine (mg)	1.33 ± 0.21	0	

Data are presented as the mean ± standard deviation or *n* (%).

In the primary outcome measure, the incidence of myoclonus in Group O was significantly lower than that in Group C (13.3% vs. 51.1%, RR = 0.45, 95% confidence interval [95%CI] = 0.310–0.667, *p* < 0.001). Moreover, compared with Group C, the incidence of myoclonus in Group O was reduced by 37.8% (Table 2).

**Table 2:** Incidence and severity of myoclonus after etomidate injection.

Variable	Group O (*n* = 45)	Group C (*n* = 45)	*p*-value
The primary outcome			
The incidence of myoclonus [*n*, (%)]	6 (13.3)	23 (51.1)	<0.001
The secondary outcome			
Severity grade [*n*, (%)]			<0.001
None	39 (86.7)	22 (48.9)	
Mild	0	4 (8.9)	
Moderate	3 (6.7)	8 (17.8)	
Severe	3 (6.7)	11 (24.4)	
Time to loss of consciousness (s)	43.24 ± 6.89	48.27 ± 10.34	0.008

Data are presented as the mean ± standard deviation or *n* (%).

In the secondary outcome measures, the severity of myoclonus in Group O was significantly lower than that in Group C (*p* < 0.001). Additionally, the time to loss of consciousness in Group O was shorter than that in Group C (43.24 s ± 6.89 vs. 48.27 s ± 10.34, *p* = 0.008).

There was no statistically significant difference in the incidence of adverse reactions such as dizziness, nausea, respiratory depression, bradycardia, hypotension, increased secretions, and coughing between the two groups (Table 3).

**Table 3:** Adverse effects in the two groups.

Variable	Group O (*n* = 45)	Group C (*n* = 45)	*p*-value
Dizziness [*n*, (%)]	0	0	>0.99
Nausea [*n*, (%)]	0	0	>0.99
Respiratory depression [*n*, (%)]	0	0	>0.99
Bradycardia [*n*, (%)]	1 (2.2)	0	1.000
Hypotension [*n*, (%)]	2 (4.4)	0	0.494
Secretions [*n*, (%)]	0	4 (8.9)	0.117
Coughing [*n*, (%)]	0	2 (4.4)	0.494

Data are presented as *n* (%).

**Figure 2:**
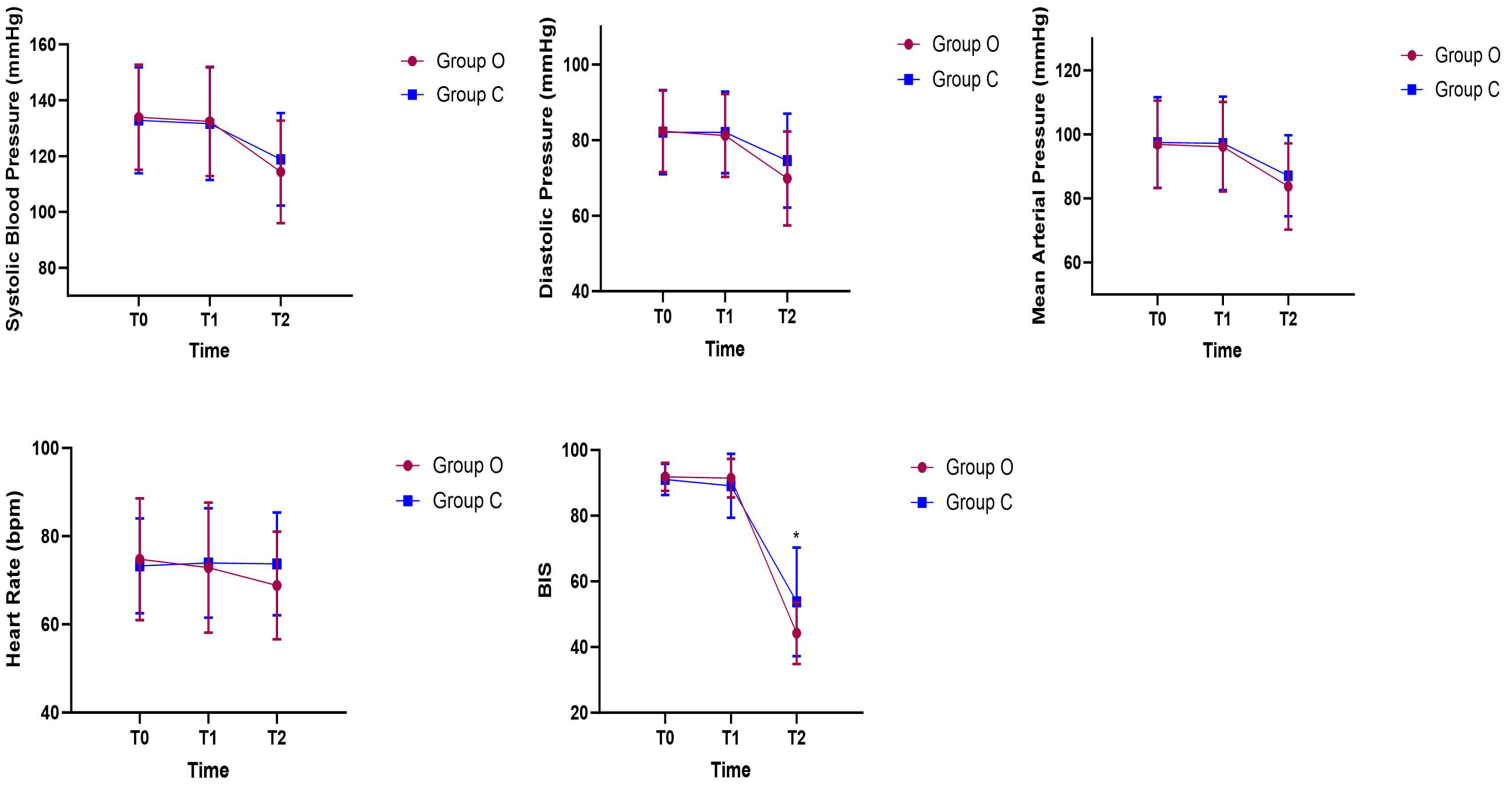
Hemodynamic changes during anesthesia induction. *p < 0.05.

There was no statistically significant difference in hemodynamic changes between the two groups during the study; however, there was a statistically significant difference in the BIS values between the two groups at time point T2 (44.23 ± 9.38 vs. 53.75 ± 16.54, *p* = 0.001) (Figure 2).

## Discussion

This study is the first to evaluate the efficacy of pretreatment with oliceridine in reducing the incidence of etomidate-induced myoclonus. The study confirmed that pretreatment with oliceridine can effectively reduce the incidence and severity of etomidate-induced myoclonus without significant adverse reactions. Furthermore, the study also found that pretreatment with oliceridine can accelerate the speed of anesthetic induction with etomidate and enhance the depth of anesthesia after induction, while having no significant impact on hemodynamics.

The mechanism by which etomidate induces myoclonus is not yet fully understood. It is hypothesized that etomidate binds to γ-aminobutyric acid (GABA) receptors, inhibiting the ascending reticular activating system in the brainstem, which subsequently suppresses higher centers such as the cerebral cortex. This suppression leads to disinhibition of lower centers, including subcortical structures, resulting in heightened sensitivity of skeletal muscle movements and the occurrence of myoclonus (11). Previous studies have shown that various drugs, including midazolam, lidocaine, dexmedetomidine, and opioids, can reduce the incidence of myoclonus to varying degrees (2, 12–14). However, recent meta-analyses comparing the efficacy of multiple drugs in mitigating etomidate-induced myoclonus suggest that pretreatment with opioids may be the most effective approach (15–17). The mechanism may involve inhibition of excitatory pathways and enhanced GABA receptor activity, thereby reducing the incidence of etomidate-induced myoclonus (18). Nevertheless, the use of opioids is also associated with a higher incidence of adverse effects (13). Previous studies have shown that fentanyl, while reducing the incidence of myoclonus, can cause significant respiratory depression (19). Additionally, pretreatment with remifentanil may lead to adverse effects such as chest wall rigidity (20). In this study, the oliceridine group did not exhibit any of the aforementioned adverse reactions during the observation period after pretreatment.

Oliceridine is a novel opioid receptor agonist. Compared with traditional opioids, it has less impact on the β-arrestin pathway, thereby reducing the incidence of opioid-related adverse reactions such as respiratory depression and gastrointestinal dysfunction (21). A study by Ma et al. compared the adverse reactions associated with the use of oliceridine and sufentanil in gastrointestinal endoscopy. The results demonstrated that oliceridine significantly reduced the incidence of respiratory depression in patients undergoing gastrointestinal endoscopic procedures (8). Additionally, Huang et al. also found that compared with traditional opioids, the incidence of opioid-related adverse reactions is lower when oliceridine is used in patient-controlled intravenous analgesia (PCIA) (22). This provides an indication that oliceridine may potentially serve as a safer alternative.

In this study, pretreatment with 0.02 mg/kg oliceridine effectively reduced the incidence of etomidate-induced myoclonus. In terms of potency, 1 mg of oliceridine is equivalent to 5 mg of morphine and also equivalent to 5 micrograms (μg) of sufentanil (23). Since there are currently few studies on the dosage of oliceridine for intraoperative anesthetic management, most studies have administered oliceridine at a low dose of 0.02 mg/kg (24–26), and research has confirmed that this dose is associated with relatively few adverse reactions. Therefore, the same dose was used in this study for observation. In this study, although the systolic blood pressure of two patients pretreated with oliceridine dropped below 90 mmHg after etomidate administration, the decrease range was within 20% of their baseline systolic blood pressure. Furthermore, this study found that patients who received etomidate alone might experience increased secretions and coughing after losing consciousness, with a wider range of BIS changes after induction; in contrast, the induction process was more stable in patients pretreated with oliceridine. However, these findings showed no statistically significant differences and are only presented for the exchange of medication experience, which warrants further research.

This study still has certain limitations. First, it is a single-center clinical study, and its findings require further confirmation by large-sample, multi-center studies. Second, this study only evaluated the effect of oliceridine pretreatment on etomidate-induced myoclonus, and did not conduct controlled trials with other opioid drugs.

## Conclusion

Pretreatment with oliceridine can significantly reduce the incidence of etomidate-induced myoclonus. Therefore, oliceridine can be used as a new pretreatment strategy when etomidate is employed for anesthetic induction.

## Data Availability

The original contributions presented in the study are included in the article/supplementary material, further inquiries can be directed to the corresponding authors.

## Footnotes

1
www.chictr.org.cn
